# Transcriptional bursts explain autosomal random monoallelic expression and affect allelic imbalance

**DOI:** 10.1371/journal.pcbi.1008772

**Published:** 2021-03-09

**Authors:** Anton J. M. Larsson, Christoph Ziegenhain, Michael Hagemann-Jensen, Björn Reinius, Tina Jacob, Tim Dalessandri, Gert-Jan Hendriks, Maria Kasper, Rickard Sandberg

**Affiliations:** 1 Department of Cell and Molecular Biology, Karolinska Institutet, Stockholm, Sweden; 2 Department of Medical Biochemistry and Biophysics, Karolinska Institutet, Stockholm, Sweden; 3 Department of Biosciences and Nutrition, Karolinska Institutet, Stockholm, Sweden; Weizmann Institute of Science, ISRAEL

## Abstract

Transcriptional bursts render substantial biological noise in cellular transcriptomes. Here, we investigated the theoretical extent of allelic expression resulting from transcriptional bursting and how it compared to the amount biallelic, monoallelic and allele-biased expression observed in single-cell RNA-sequencing (scRNA-seq) data. We found that transcriptional bursting can explain the allelic expression patterns observed in single cells, including the frequent observations of autosomal monoallelic gene expression. Importantly, we identified that the burst frequency largely determined the fraction of cells with monoallelic expression, whereas the burst size had little effect on monoallelic observations. The high consistency between the bursting model predictions and scRNA-seq observations made it possible to assess the heterogeneity of a group of cells as their deviation in allelic observations from the expected. Finally, both burst frequency and size contributed to allelic imbalance observations and reinforced that studies of allelic imbalance can be confounded from the inherent noise in transcriptional bursting. Altogether, we demonstrate that allele-level transcriptional bursting renders widespread, although predictable, amounts of monoallelic and biallelic expression in single cells and cell populations.

## Author summary

Genes are transcribed into RNA and further translated into proteins. The maternal and paternal copy of each gene are typically transcribed independently, and transcription itself occur in discrete stochastic bursts (transcriptional bursts). Pioneering single-cell analysis of RNA across cells revealed abundant fluctuations in the amounts of maternal and paternal RNA in cells, with frequent observations of RNA from only the maternal or paternal gene copy (monoallelic expression). In this study, we investigated to which extent the observed monoallelic expression across single cells can be explained by transcriptional bursting. We demonstrate that the process of transcriptional bursting is sufficient to explain the amount of monoallelic expression, and we further demonstrate that the frequency of bursts mainly determines the frequency of monoallelic observations. Furthermore, we show that transcriptional bursts may lead to false positive observations of monoallelic expression across cell populations. Therefore, stochastic transcription renders large fluctuations in allelic origin of RNA in cells over time, including frequent monoallelic observations when profiling single cells.

## Introduction

Stochastic transcription generates biological variation across individual cells of the same cell type [1,2]. Independent transcriptional bursting of each allele [1,3–6] generates periodic fluctuations in the abundance of transcripts, and unequal expression of two functionally different alleles can give rise to cellular and phenotypic variability [7].

As single-cell RNA-sequencing (scRNA-seq) protocols arrived at higher sensitivity and accuracy [8–10], it has become feasible to study transcriptome-wide patterns of allelic expression across single cells. Indeed, allele-sensitive scRNA-seq analysis across individual cells have revealed that RNA from substantial numbers of autosomal genes were detected from only a single allele in individual cells at any given time point [11]. The observed autosomal random monoallelic expression (aRME) could be generated from transcriptional bursting [3,4,6,12], in particular since subsequent work demonstrated that the allelic patterns were primarily due to a stochastic process in somatic cells, rather than a mitotically heritable characteristic [13]. Furthermore, allele-specific RNA FISH of autosomal genes *in situ* has shown that transcriptional bursting can explain the observed aRME of individual genes [14]. However, the explicit relationship between aRME and transcriptional burst kinetics has not been systematically explored.

**Fig 1:**
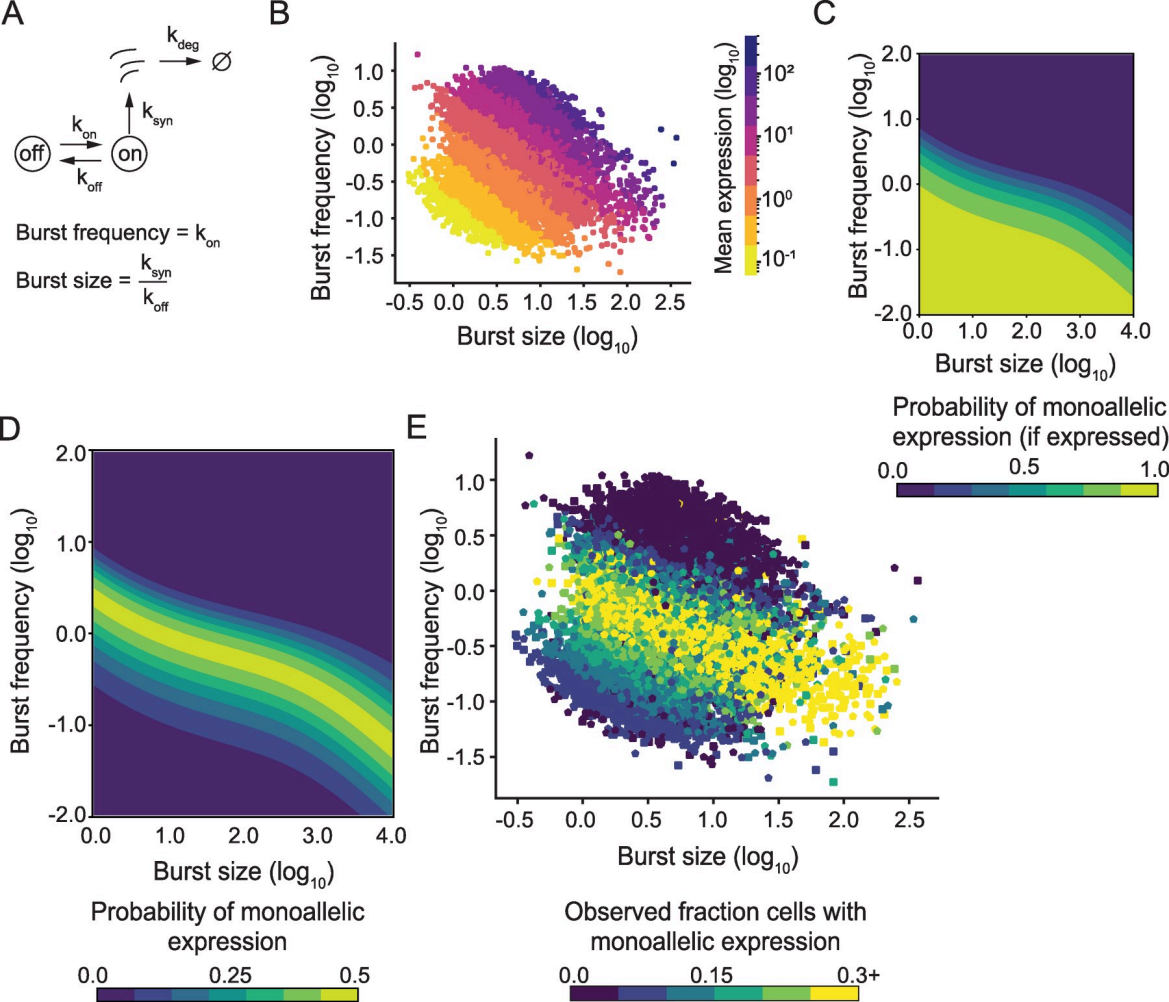
The theoretical effect of transcriptional bursting on dynamic random monoallelic expression. (A) Illustration of the model used for transcriptional burst kinetics. The time for the gene to transition are given by the exponentially distributed parameters k_*on*_ (from off to on) and k_*off*_ (from on to off). While the gene is active, the gene is transcribed at rate k_*syn*_. The burst frequency is given by k_*on*_ and the average number of transcripts produced in a burst (burst size) is given by k_*syn*_ /k_*off*_. (B) A scatter plot showing burst frequency and burst size estimates from the C57 allele of autosomal genes in mouse fibroblasts (CAST/EiJ × C57BL/6J, *n* = 7,606 genes), where each gene is colored based on the mean expression level of that gene (mean number of observed UMIs per cell). (C) Contour plot of the conditional probability of observing monoallelic expression when there is expression of that gene in the parameter space of burst frequency and size. (D) Contour plot of the probability of observing monoallelic expression in the parameter space of burst frequency and size, irrespectively if the gene is expressed or not. (E) A scatter plot showing burst frequency and burst size estimates from both alleles in mouse fibroblasts (C57 square, CAST pentagon, *n* = 7,606 autosomal genes), where each gene is colored based on the fraction of cells which expressed the gene monoallelically from that allele (*n* = 682 cells).

Analysis of transcriptional burst kinetics is generally based on the two-state model of transcription [4,15] (Fig 1A), which is the simplest model to describe both bursting and constitutive expression dynamics, and it has been extensively used to investigate quantitative relationships between burst kinetics and gene-level measurements [4,5,16]. The two-state model consists of four allele-specific parameters that may accommodate different transcriptional kinetics, mainly characterized by the burst frequency and size, with frequency normalized by mRNA degradation rates. A severe limitation to investigating the general implications of transcriptional bursting in diploid cells has been the challenge of obtain reliable allelic estimates of transcriptional burst kinetics for sufficiently large numbers of genes. However, this barrier was recently overcome by advances in the inference of transcriptional burst kinetics from allele-sensitive scRNA-seq [6,16,17], culminating in the demonstration that enhancers drive burst frequencies and that core promoter elements affect burst size [6].

In the present study, we used state-of-the-art scRNA-seq measurements to infer transcriptional bursting parameters transcriptome-wide across cells of a mouse cross breed (CAST/EiJ × C57BL/6J). We show that the observed allelic expression patterns across cells are consistent with those predicted from the inferred transcriptional bursting parameters, explaining the frequent observations of monoallelic expression in single-cell data [11,13] as independent bursts of transcription from each allele. We further show, for *in vitro* and *in vivo* cells, that the fraction of monoallelic expression is mainly driven by the frequency of transcriptional bursts rather than burst sizes, whereas allelic imbalance is a consequence of both burst frequencies and size.

## Results

We first investigated the theoretical impact of transcriptional burst kinetics on random monoallelic gene expression, using the two-state model of transcription (Fig 1A) that consists of the parameters (k_*on*_,k_*off*_,k_*syn*_) which describe the distribution of transcripts at steady state (Methods). The same mean expression level across cells can result from multiple distinct combinations of burst frequencies and sizes, which is readily observable in scRNA-seq data [6](Fig 1B). We examined how the probability of observing monoallelic gene expression in cells depend upon the transcriptional bursting parameters. To this end, we modelled transcriptional bursting processes for two alleles with identical kinetics as a function of burst frequency and size throughout the transcriptional bursting space. The probability of detecting *n* RNA transcripts from one allele at a given time can be expressed as *P*(*n*|k_*on*_,k_*off*_,k_*syn*_). By conditioning the probability on the total probability of expression *P*(*monoallelic*|*expressed*), we find that genes with low burst frequency (k_*on*_) and size (k_*syn*_/k_*off*_) are always monoallelically expressed given that there is expression at all (Fig 1C). A combination of high burst frequency and size gives exclusively rise to biallelic states, while intermediate combinations of these extremes lie on a spectrum in-between. If we did not condition on expression, we observed a ridge of states of monoallelic expression where biallelic and no expression dominate on either side of the ridge respectively (Fig 1D).

We next generated allele-resolution scRNA-seq data from 682 individual primary mouse fibroblasts (F1 offspring of CAST/EiJ and C57BL/6J crosses) using Smart-seq3[10], the scRNA-seq method that currently that has highest sensitivity and best coverage across genes. The deeply sequenced cells (average of 3.5M read pairs per cell) resulted in the average detection of 206,944 molecules per cell (i.e. error corrected UMIs). We inferred transcriptional burst kinetic parameters from the molecule counts observed per gene and allele (S1 Table), as described previously [6], which resulted in robust transcriptional burst inference for both alleles independently for 7,606 autosomal genes. Using these data, we asked to what extent the measurements of monoallelic and biallelic expression from scRNA-seq experiments concur with the two-state model predictions. Strikingly, the observed fraction of monoallelic expression per gene was highest on the ridge that was visible across the parameter space of burst kinetics (Fig 1E), as predicted by the theory (Fig 1D).

**Fig 2:**
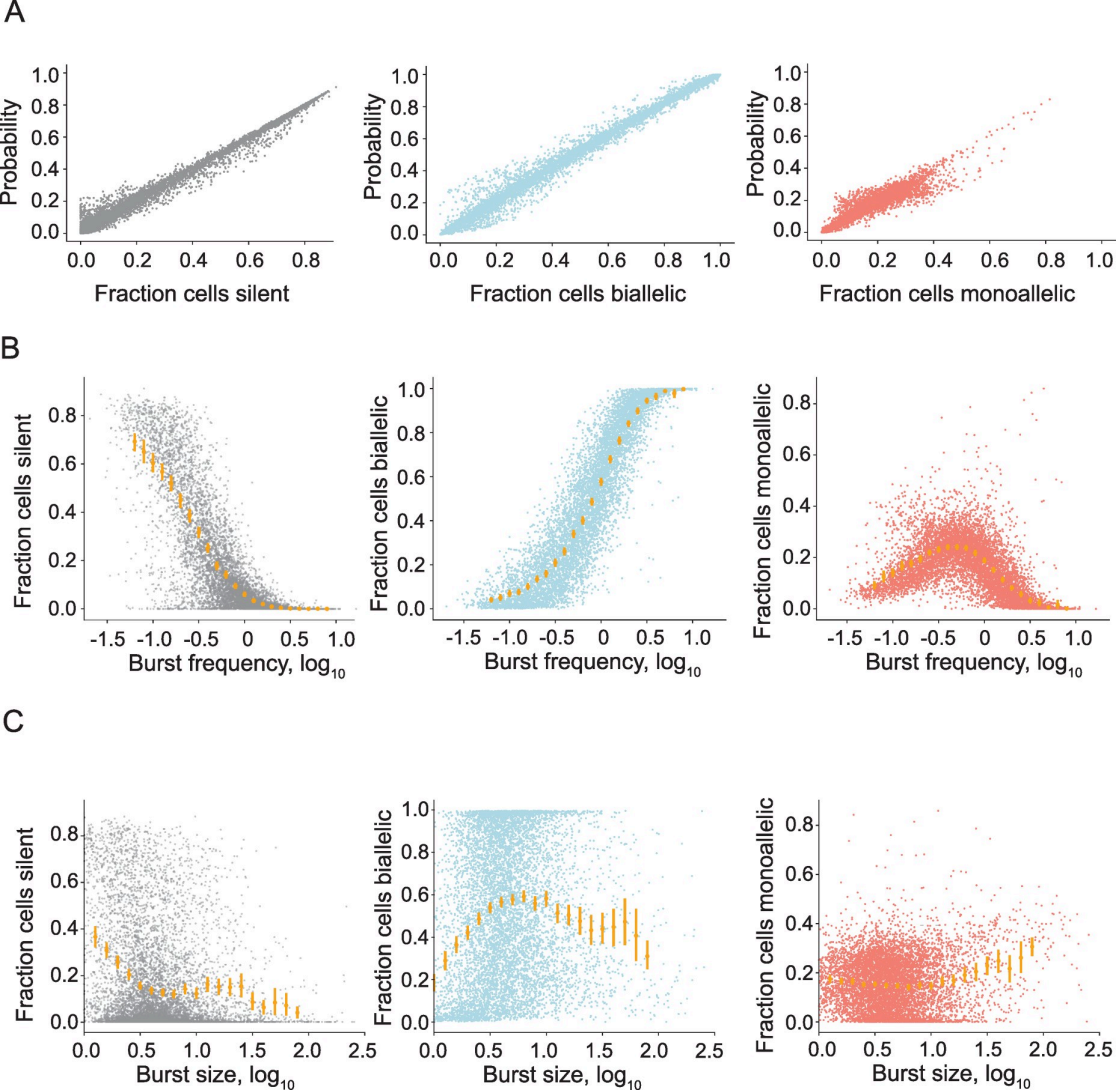
The relationship between transcriptional burst kinetics and dynamic random monoallelic expression in primary mouse fibroblasts. (A) Correlations between the predicted and observed fraction of cells with: no expression (left), biallelic expression (middle) and monoallelic expression from the C57 allele (right), *n* = 7,606 genes. (B) The observed fraction of cells with silent (right), biallelic (middle), and monoallelic (C57, right) compared to burst frequency for 7,606 autosomal genes inferred in mouse fibroblasts. (C) The observed fraction of cells with silent (right), biallelic (middle), and monoallelic (C57, right) compared to burst size for 7,606 autosomal genes inferred in mouse fibroblasts.

We then continued the comparison between predicted patterns of allelic expression to those observed in the scRNA-seq data, by estimating the probabilities of observing a cell which is either silent, biallelic, monoallelic on CAST or monoallelic on C57 for all genes based on their bursting parameters (S2 Table), assuming that transcription occurs independently on each allele. The predicted fractions of cells in each state were highly correlated with the observed fraction of cells in each category (Fig 2A and Table 1) demonstrating that modelling transcription using the two-state model at each allele independently agrees with experimental allelic expression analyses by scRNA-seq. We also performed cross-validation so that the bursting kinetics were inferred from a subset of cells and the remaining cells were used to estimate fraction of allelic observations in cells, which reassured that we were not overfitting the model (Table 1). We also investigated the potential agreement between theory and observations if we were to model the data using the simpler Poisson distribution. The Poisson model predictions resulted in grossly overestimating biallelic expression and underestimating the fractions of no expression (S1 Fig), demonstrating significantly worse fit than the bursting model. The theoretical results indicated levels of observed autosomal monoallelic gene expression can result from modulation of either burst frequency or size. To investigate whether either of the parameters was the more determining parameter for the amount of monoallelic expression observations, we examined the profile of burst frequency and size in relation to monoallelic expression to isolate their relative contributions. Comparing the burst frequency to the observed fraction of monoallelic expression showed a striking relationship (Fig 2B). At lower burst frequencies, we observed very low amounts of monoallelic expression. The fraction of monoallelic expression increased as the burst frequency was elevated, up until the point where biallelic expression became the predominant observation and monoallelic expression declined. This relationship was also clear in the theoretically predicted case which demonstrated that our model predictions were consistent with the biological data (S2 Fig). The same analysis on burst size showed that the distribution of monoallelic expression was almost uniform over burst size with a tendency of genes with large burst sizes to have more biallelic expression (Fig 2C). Therefore, while burst size has the theoretical capability to influence the amount of monoallelic expression in cells, it plays a minor role relative to burst frequency. The predominant role of burst frequencies in determining monoallelic gene expression can also be seen in the slope of the ridge of monoallelic expression (Fig 1D and 1E).

**Table 1:** Spearman correlation coefficients for predicted and actual fraction of cells in each category over genes.

	No Expression	Biallelic	Monoallelic (CAST)	Monoallelic (C57)
**Full data**	0.99	0.97	0.96	0.96
**Train/Test Cross-validation**	0.98	0.97	0.88	0.89

Full data uses all the cells to infer transcriptional burst kinetics compared to all cells. The cross-validation approach randomly splits the cells into two equal groups. One group is used to infer kinetics for prediction and the other group to calculate the observed fraction.

**Fig 3:**
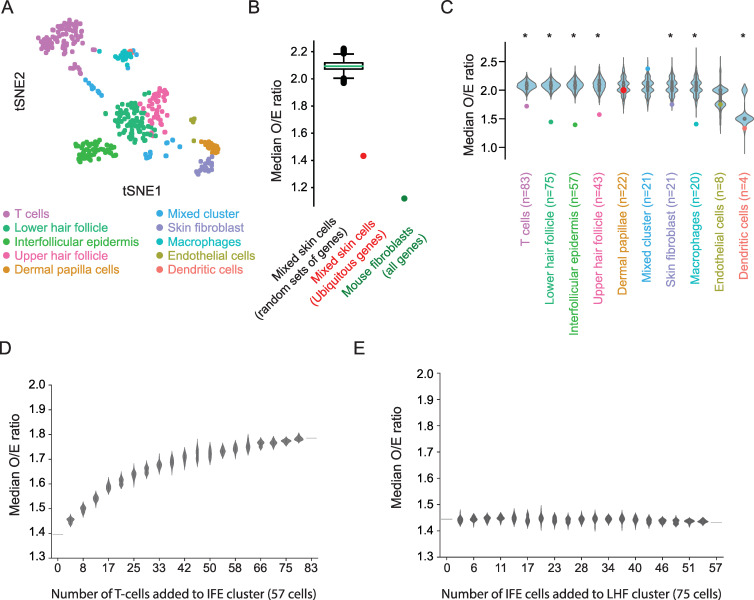
Heterogeneity in cell clusters from an *in vivo* experiment in mouse skin measured by observed-to-expected biallelic expression. (A) T-distributed stochastic neighbour embedding (tSNE) of the skin cells, colored by SNN-based clustering (*n* = 354 cells). (B) The median observed-to-expected (O/E) ratio of biallelic expression, comparing the theoretical predictions from burst kinetics to that observed in all cells without stratifying cells to clusters. Boxplot show median O/E biallelic expression from random sets of genes (*n* = 3,727 autosomal genes and 100,000 permutations) whereas the red dot show the O/E ratio when analyzing ubiquitously expressed genes in all cells. For comparison, the analyses of all genes in primary fibroblasts are shown in green. (C) The median O/E ratio of biallelic expression within cell clusters shown as colored dots. These were compared to randomly selected cells of the same size (*n* = 83, 75, 57, 43, 22, 21, 21, 20, 8, 4 cells respectively, 1,000 permutations for each cluster). Asterisk denotes significance at alpha = 0.05. (D) The median O/E ratio after adding *n* number of cells from the T-cell cluster to the Interfollicular epidermis (IFE) cluster. Bootstrapped 20 times. (E) The median O/E ratio after adding *n* number of cells from the Interfollicular epidermis (IFE) cluster to the Lower hair follicle (LHF) cluster. Bootstrapped 20 times.

To extend the inference and analyses of transcriptional burst kinetics to cell types *in vivo*, we sequenced individual cells from dorsal skin of the same mouse cross breed (C57BL/6JxCAST/EiJ) using Smart-seq2 [18]. We analyzed the 354 single-cell transcriptomes that passed quality control filtering, and those could be grouped into 10 clusters (Fig 3A) that could be further assigned to cell types of variable heterogeneity using existing skin single-cell transcriptomics data [19]. The relationship between burst kinetics and random monoallelic gene expression for cells *in vivo* was consistent with the data from primary fibroblasts (S3 Fig), reinforcing the generality of our results to cells *in vivo*. For the application of the steady state distribution of the two-state model we assume ergodicity [20]. In scRNA-seq experiments, large numbers of individual cells are sampled to statistically characterize what the process would look like if we followed one cell over time, with the underlying assumption that sampled cells follow similar bursting kinetics. Due to the heterogeneous cellular composition of certain clusters, we therefore wanted to quantify how well each cell-type cluster represent the same underlying bursting process. We therefore assessed the extent to which a cell cluster predicted its own biallelic expression based on the model of independent allelic transcriptional bursting (Methods). We anticipated that high heterogeneity within a cell cluster would show an underestimation of predicted biallelic expression due to subsets of heterogeneous cells with higher burst frequency for certain genes. Indeed, the median observed-to-expected ratio of biallelic expression (O/E ratio) based on all cells (irrespective of clustering) indicated a clear transcriptome-wide underestimation of biallelic expression (median = 2.1, *n* = 10,543 genes). To examine the potential of allelic-expression modelling as an unbiased method to assess the degree of bursting heterogeneity within groups of cells, we first examined ubiquitously expressed genes as they are expected to have less cell-type-specific transcriptional burst regulation compared to other genes and thereby have observed biallelic observations closer to the expected value (an O/E ratio closer to 1). Indeed, these genes had a significantly lower O/E ratio compared to randomly selected subsets of genes and were close to the ratio observed in the fibroblast cells, which show high bursting homogeneity according to this metric for all genes (*P* < 10^−5^, permutation test, Fig 3B). This result was not biased due to total expression level, as evaluated against a set of random genes with similar expression levels (S4 Fig). Importantly, the stratification of cells into clusters greatly improved the O/E ratio compared to randomly selected sets of cells, with the exception of three clusters (containing mixed unassigned cells, endothelial and dermal papillae cells, *P* < 10^−3^, permutation test, Fig 3C). By artificially adding cells from one cluster to another cell clusters, we grossly evaluated the sensitivity of this metric. When adding cells from the T-cell cluster to the cluster of interfollicular epidermis (two dissimilar clusters), the median O/E ratio increased rapidly with the addition of only a few T-cells (Fig 3D), whereas adding interfollicular epidermis cells to the cluster of lower hair follicle cells (two similar clusters) resulted in no detectable increase in the median O/E ratio (Fig 3E). Therefore, in the analyzed cells the observed-to-expected biallelic expression metric could quantify larger heterogeneity in cell clusters without having the resolution to assess purity among cell types with more similar transcriptomes.

**Fig 4:**
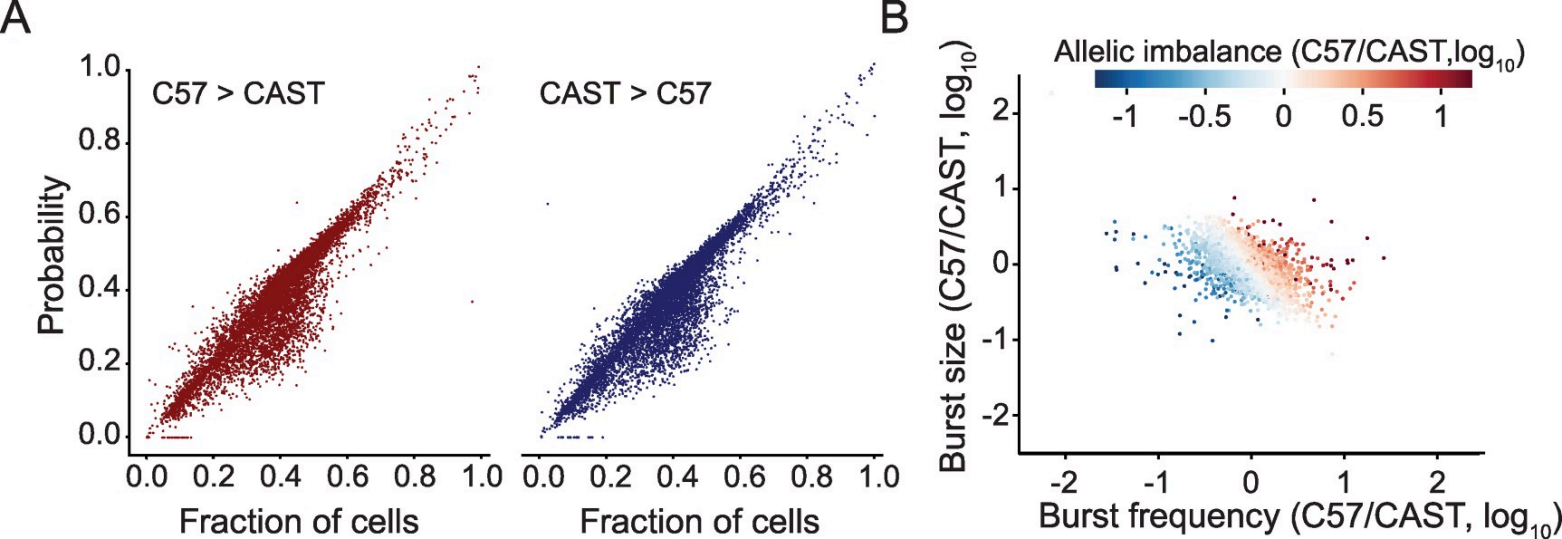
Allelic bias is affected by relative changes in both burst frequency and size. (A) Comparison between the probability of observing allelic imbalance between the alleles and the actual fraction of cells with the imbalance (*n* = 7,606 autosomal genes). (B) The relative allelic differences in burst kinetics for each gene, colored by their allelic bias (*n* = 7,606 genes).

Investigating gene expression at the discrete level of monoallelic and biallelic expression was motivated by their frequent occurrence in single-cell data. We naturally extended these analyses to the whole range of biased expression between the alleles, defined here as the theoretical probabilities of one allele occurring in larger or equal amounts to the other allele, *P*(C57 > CAST), *P*(CAST > C57) and *P*(CAST = C57). We estimated these probabilities in the primary fibroblasts data generated with Smart-seq3. Most genes have very similar kinetics between the two alleles and therefore a close to equal probability of unequal expression for each allele, as measured by *P*(C57 > CAST | C57 ≠ CAST) (S5A Fig). The probability of equal expression is dominated by the outcome of no expression on either allele, which is predictably related to the burst frequencies of the two alleles of the gene (CAST = C57 = 0, S5B Fig). The probabilities were in good agreement with the observed fractions of allelic bias (Fig 4A). By comparing the fold changes in burst size and frequency between alleles to their observed fraction of allelic bias, we found that the relative differences in transcriptional burst kinetics in burst frequency as well as size tended to affect allelic bias for that gene (Fig 4B). Interestingly, simultaneous relative changes in both burst frequency and size may cancel each other out. For example, a reduction in burst size may be compensated by an increase in burst frequency (Fig 4B; visualized along the diagonal of the scatter plot). By using linear regression with allelic bias as the dependent variable, we determined that relative changes in both burst frequency and size together explain the allelic bias to a high degree (*R*^2^ = 83.7%) and both relative changes have significant impact on allelic bias (Table 2).

**Table 2:** Ordinary least squares regression results for the effect of burst kinetics on allelic imbalance.

	coef	std err	t	P>|t|	[0.025	0.975]
**Intercept**	-0.0017	0.001	-1.278	0.201	-0.004	0.001
$log_{10}(\frac{bf_{C57}}{bf_{CAST}})$	1.2864	0.007	175.109	0.000	1.272	1.301
$log_{10}(\frac{bs_{C57}}{bs_{CAST}})$	0.9272	0.008	120.609	0.000	0.912	0.942
$log_{10}\left(\frac{bf_{C57}}{bf_{CAST}}\right):\;log_{10}(\frac{bs_{C57}}{bs_{CAST}})$	-0.740	0.021	-3.593	0.000	-0.114	-0.034

Dependent variable: $\log_{10}\frac{C57>CAST}{CAST>C57}$, R-squared: 0.805. bf: burst frequency, bs: burst size,coef: linear regression coefficient, std err: standard error, t: t-statistic.

**Fig 5:**
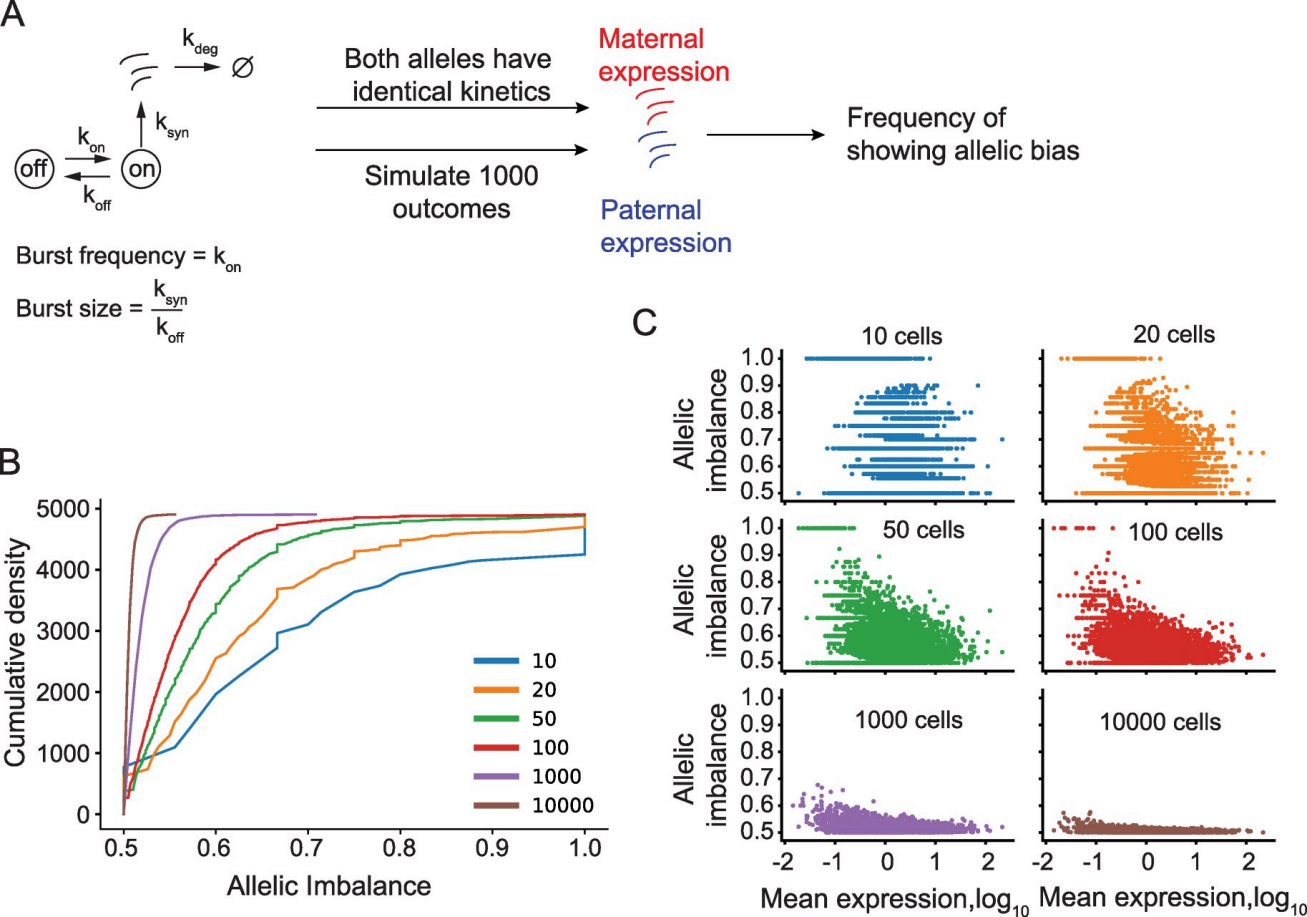
Low-expressed genes frequently show false positive allelic imbalance due to transcriptional bursting. (A) Outline of the simulation strategy. (B) The cumulative distribution of allelic bias of the simulated genes with the same kinetics (*n* = 4,905 autosomal genes), where allele with the highest allelic bias is the chosen value for each gene. (C) The relationship between the mean expression of a gene and allelic bias based on the number of simulated cells (*n* = 4,905 genes). Figure based on data from [6].

To determine the extent to which transcriptional bursting may give rise to false positives in studies of allelic imbalance in cell populations, we simulated the expression from two alleles with kinetics identical to those inferred from the C57 allele for different number of cells (Fig 5A, *n* = 10, 20, 50, 100, 1,000 and 10,000 cells). We then estimated the allelic imbalance for all genes in the bulk population based on a model that expected equal expression from both alleles. At a low number of pooled sequenced cells, the variance in expression due to transcriptional bursting severely impacts the allelic imbalance measurements and give rise to a high number of false positives, but becomes increasingly stable with a higher number of cells (Fig 5B). In relation to mean expression, we find that it is only for low-expressed genes that false positive allelic imbalance becomes frequent, and this declines as the number of cells increases (Fig 5C).

## Discussion

In this study, we explored to what extent transcriptional bursting can explain the patterns of random monoallelic gene expression of autosomal genes observed in single-cell analysis [7]. We report a striking agreement between the two-state model and biological observations of cellular allelic expression patterns, and frequencies of cells with monoallelic and biallelic expression closely follows the frequencies predicted from theory. Thus, transcriptional bursting result in extensive monoallelic expression of autosomal genes that explains earlier observations of frequent monoallelic expression of autosomal genes in scRNA-seq data[11,13]. Moreover, we show that burst frequency largely determines how often a gene is monoallelically expressed in somatic diploid cells.

We also explored to what extent transcriptional bursting can lead to spurious observations of allelic imbalance in cell population studies. In full agreement with single-molecule RNA FISH analyses of allelic gene expression in cells *in vivo* [14], we found that lowly expressed genes can be falsely identified as having allelic imbalance simply due to their stochastic transcription. It is interesting in this context to note that most of the previously identified genes with fixed autosomal random monoallelic expression were detected at very low levels around two RNA transcripts per cell on average when expressed [13,21,22]. It is clear that future studies of monoallelic gene expression and allelic imbalance in diploid cells need to consider the consequences of transcriptional bursting in order not to attribute stochastic fluctuations as regulated allele-specific expression.

Transcriptional bursting results in considerable cellular heterogeneity from the unequal expression of two functionally different alleles (e.g. see [23]). It is however not explored to what extent such variation has phenotypic consequences. The relative abundances of protein products resulting from translation of the two different alleles may be affected by burst size which could be relevant in the case of phenotypes that result due to the stoichiometric constraints present in signaling pathways and gene networks. Interestingly, transcriptional bursting was recently shown to impact T-cell linage commitment [24], which raises the intriguing question whether cell fate decisions in general could be affected by stochastic transcription. Since burst frequency is preferentially encoded in enhancer regions [6] it is likely that mutations in *cis*-regulatory sequences or trans-activating factors may affect the penetrance of phenotypes. This may be particularly relevant in the case of lineage commitment, which exhibits switch-like irreversible activation.

Inference of transcriptional burst kinetic parameters from the single-cell observations of a large number of cells, rely on the cells being homogeneous, i.e. that the cells have very similar bursting kinetics. We show that groups of cells that are more heterogeneous are characterized by deviations in the expected patterns of allelic expression from theory. There is currently great excitement in using single-cell RNA-sequencing to identify and characterize cell types, sub-types and cellular states throughout human tissues and in model organisms [25], and computational strategies to assess cell cluster accuracy would be very useful. Here, we explored to what extent deviations in biallelic expression from the predicted, where heterogeneous groups of cells have biallelic expression at a much higher frequency than what would be predicted (and therefore a higher biallelic O/E ratio). Although this strategy had power to assess purity of clusters from cells of different types, we found that the allelic modeling had low power to assess heterogeneity between closely related cell types. A future strategy could be to assign cells to a latent space of bursting parameters governing the random process by which molecules arise, so that the assumption of homogeneity to study transcriptional bursts can be effectively bypassed.

Together, we have explored how transcriptional bursting through the two-state model predicts the observed amounts of allelic expression patterns in mammalian cells, finding remarkable agreement between predictions and observations. Therefore, the generalized theoretical framework of bursting combined with transcriptome-wide kinetic parameters has important implications to the interpretation of allele-specific gene expression in cells, and ultimately, to our understanding of phenotypic variation in diploid organisms.

## Methods

### Ethics statement

The research carried out in this study was approved by the Swedish Board of Agriculture (Jordbruksverket: N95/15).

### Generation of Smart-seq3 libraries

Smart-seq3 libraries were generated according to previously published protocol [10]. Briefly, primary mouse fibroblasts were obtained from tail explants of CAST/EiJ × C57/Bl6J mice (>10 weeks old) and passaged for at least ten days. Cells were sorted in 384-well plates with dead-cell exclusion (propidium iodide; Thermo Fisher) on a FACSMelody (BDBiosciences) using a 100 μM nozzle. Plates contained 3 μl of Smart-seq3 lysis buffer (6.67% PEG (Sigma), 0.10% Triton X-100 (Sigma), 0.5 U L-1 of recombinant RNase inhibitor, (Takara), 0.67 M Smart-seq3 oligo-dT primer (5-biotin-ACGAGCATCAGCAGCATACGA-T30VN-3; IDT), 0.67 mM dNTPs (Thermo Scientific)) and were spun down and stored at 80°C immediately after sorting. The standard Smart-seq3 protocol was applied, using 20 cycles of PCR for pre-amplification of cDNA, a 0.6:1 bead:sample ratio for purification of pre-amplified cDNA (using homemade 22% PEG beads) and tagmentation of 100 pg purified cDNA using 0.1 μL of ATM. Libraries were indexed using 12 cycles of PCR for library amplification of the tagmented samples using custom-designed Nextera index primers containing 10-bp indexes and 5’ phosphorylation. Samples were finally pooled, bead purified at a ratio of 0.7:1 (using homemade 22% PEG beads) and prepared for sequencing on a DNBSEQ-G400RS (MGI) generating 100 bp paired-end reads.

### Analysis of Smart-seq3 libraries

Fastq files were processed using zUMIs v2.9.3e [26] with STAR v2.7.3a [27] to map reads to the mouse genome (mm10) and generate error-corrected UMI count tables for Ensembl gene annotations (GRCm38.91). UMI counts were classified into the two alleles by analyzing coverage over validated heterozygous SNP positions (see https://github.com/sandberg-lab/Smart-seq3/tree/master/allele_level_expression for details).

### The two-state (beta-poisson) model

The model used for stochastic gene expression is a particular case of a birth-and-death process in a Markovian environment. In short, the model has the states (*i*, *n*) with *i* being 0 or 1 indicating if the gene is active or not, and *n* is the number of RNA transcripts in the cell.

In the off state, the gene can turn on with the rate k_*on*_. In the on state, the gene can turn off with rate k_*off*_ and produce one RNA transcript with the rate k_*syn*_. Regardless of the state, one RNA transcript can be degraded with rate λ. At the steady state of this process, the stationary distribution can be shown to be described by the Poisson-beta distribution, in which we let

$$p|k_{on,}k_{off}∼\mathrm{Beta}\left(k_{on},\;k_{off}\right)$$

$$n|k_{syn},\;p∼\mathrm{Poisson}\left(p\;k_{syn}\right)$$

The resulting marginal distribution *P*(*n*| k_*on*_, k_*off*_, k_*syn*_) is the probability distribution for the amount of RNA transcripts observed at steady state given the rates k_*on*_, k_*off*_, k_*syn*_.

### The Poisson model

We compared the two-state model to the Poisson model. For the Poisson model, we used the mean number of molecules as the estimator for the λ parameter where *n* | λ ~ Poisson(λ).

### Inference of transcriptional burst kinetics

We calculated the number of molecules per allele by first calculating the fraction of reads supporting the CAST allele and multiplying that by the total number of UMIs present in that cell and gene. The remaining fraction of UMIs were assigned as C57. However, we did not link the UMI to its genotype. UMI counts but no allele supporting reads were considered as missing data. We inferred kinetic bursting parameters for 9,337 and 9,606 genes for the C57 and CAST allele respectively from 682 F1 cross-breed (CASTxC57) adult tail fibroblasts. The intersection of kinetic parameters between both alleles resulted in 7,606 usable genes for our analysis. The method to infer these parameters given allele-sensitive scRNA-seq data is described in [6] and the code for doing so is available at (https://github.com/sandberg-lab/txburst), and we required genes to have confidence interval spans (CI-high / CI-low) below 10 for both burst size and frequency.

### Calculating the probabilities and observed fractions for silent, biallelic, monoallelic expression and allelic bias

From the transcriptional burst kinetic parameters, we can calculate the probability of an allele expressing a given gene or not at the time of sampling. We define a function of the probability of observing *k* UMI counts for an allele of gene *g* given the parameters, *P*(*K* = *k*| k_*on*_, k_*off*_, k_*syn*_).

With the resulting genes we can calculate the probabilities of an allele of a gene not being expressed, i.e. *P*_gC57_ = *P*_gC57_ (*K* = 0| k_*on*_, k_*off*_, k_*syn*_) and *P*_gCAST_ = *P*_gCAST_ (*K* = 0| k_*on*_, k_*off*_, k_*syn*_).

This allows us to calculate the probabilities of:

Probability of no expression on any allele: *P*_*silent*_ = *P*_gC57_ *P*_gCAST_

Probability of monoallelic expression on the C57 allele: *P*_*monoC57*_ = (1—*P*_gC57_) *P*_gCAST_

Probability of monoallelic expression on the CAST allele: *P*_*monoCAST*_ = (1—*P*_gCAST_) *P*_gC57_

Probability of biallelic expression: *Pbiallelic* = (1—*P*_gC57_) (1—*P*_gCAST_)

These probabilities assume that the alleles burst independently, which previous analysis have indicated (see [11]). Moreover, the computed probabilities closely followed the observed fractions, giving further support to this assumption. The contour plots in Fig 1C and 1D are based on 100x100 parameter combinations where k_*off*_ is varied to change burst size while *k*_*syn*_ is held constant at 100.

For each gene, we calculated the fraction of no expression, monoallelic on C57, monoallelic on CAST and biallelic expression by averaging the following conditional statements over the cells where *n*_*allele*_ refers to the number of actual UMI counts for that allele in that cell:

No expression: *n*_*C57*_ = 0 and *n*_*CAST*_ = 0

Monoallelic expression on the C57 allele: *n*_*C57*_ > 0 and *n*_*CAST*_ = 0

Monoallelic expression on the CAST allele: *n*_*C57*_ = 0 and *n*_*CAST*_ > 0

Biallelic expression: *n*_*C57*_ > 0 and *n*_*CAST*_ > 0

For the comparisons between predicted and observed values we used spearman correlations.

We then calculated the theoretical probabilities of the allele of a gene occurring in larger amounts than the other allele by considering the probability of

$$P(a_{1}>a_{2})=\sum_{k=0}^{n}P(a_{1}>a_{2}|a_{1}=k)P(a_{1}=k)$$

where a_1_ and a_2_ is the number of RNA transcripts from allele 1 and 2 respectively and *n* is the highest number of RNA transcripts for a_1_ with a non-zero probability of being observed. For each gene we then find three probabilities *P*(C57 > CAST), *P*(CAST > C57) and *P*(CAST = C57). The code is available on Github (https://github.com/sandberg-lab/aRME_and_bursting).

### Cross-validation of predictions

To assess whether the above predictions hold in general, we generated a test and train dataset by randomly splitting the cells into two equal groups. We inferred the kinetics and predicted using the train set, and we compared that to the observed allelic expression detected in the test set.

### Preparation and sequencing of skin cells

Skin tissue was dissected from 9 week old female F1 offspring of matings between CAST/EiJ and C57BL/6J mice (approval by the Swedish Board of Agriculture, Jordbruksverket: N95/15). Cells were dissociated from skin as described in Joost et al. [19], or using GentleMACS (Miltenyi Biotec); with both methods giving similar cell yields and viability. Briefly, for the GentleMACS method, dorsal skin was cut and minced into small pieces (approximately 1x1mm) and incubated in HBSS (Sigma) + 0.04% BSA (Sigma) + 0.2% Collagenase Ia (Sigma) at 37°C for 60 minutes with occasional agitation. Thereafter this slurry was processed on a GentleMACS (Miltenyi Biotec) with 2x Program D, cell-strained (70um) and washed. Residual tissue was further treated with HBSS + 0.05% Trypsin-EDTA (Sigma) at 37°C for 15 minutes and processed likewise. For the Joost et al method [19], GentleMACS dissociation was substituted with manual disaggregation by smashing tissue fragments against a cell strainer with the piston from a 5 mL syringe. Cells were sorted into 384-well plate by FACS, and subject to Smart-seq2 single-cell RNA-sequencing library creation [18]. The single-cell libraries were sequenced on an Illumina HiSeq4000, the sequence fragments aligned to the mouse genome (mm10) and summarized into expression levels (RPKMs) and allele-resolved expression, as previously described [6].

### Analysis of skin cells

Single-cell data was processed and analysed using Seurat (version 2.3.4), including log-normalization, regression of the total number of detected reads, identification of genes with most biological variation (n = 1,000), SNN-based clustering (distances in PCA-space, using the 20 top principal components), followed by manual curation of certain clusters (endothelial cells, dermal papillae, dendritic cells, mixed cluster). Cells with less than 100k mapped reads were excluded from the analysis (30 cells). The allelic expression levels were used for transcriptional burst kinetics inference, as described above. The discrepancy in scale between burst size values inferred from Smart-seq2 data and Smart-seq3 data is due to UMIs, for a more detailed discussion see [6].

### Assessing heterogeneity of cell-type clusters by observed-to-expected biallelic expression

To calculate the observed-to-expected ratio of biallelic expression for each gene, we calculated the expected fraction of cells with biallelic expression based on the model of independent bursts of transcription,

$$E_{biallelic}(g)=\frac{1}{C^{2}}\sum_{k}^{C}I(n_{k,C57})\sum_{k}^{C}I(n_{k,CAST})$$

where *C* is the number of cells, *k* the *kth* cell and *I(n)* is the indicator function

$$I(n)=\left\{ \begin{array}{l} 1,\;n>0\\ 0,\;n=0 \end{array}\right.$$

We then calculate the observed number of cells with biallelic expression for that gene, O_biallelic_(g), to combine them to obtain O_biallelic_(g)/ E_biallelic_(g). The list of ubiquitously expressed genes was obtained from [28].

### Ordinary least squares regression of the effect of burst kinetics on allelic bias

We used the OLS module of the statsmodels package in Python with the formula:

$$\log_{10}\frac{C57>CAST}{CAST>C57}=\beta_{1}\log_{10}\frac{bf_{C57}}{bf_{CAST}}+\beta_{2}\log_{10}\frac{bs_{C57}}{bs_{CAST}}+\beta_{3}\log_{10}\frac{bf_{C57}}{bf_{CAST}}\cdot \log_{10}\frac{bs_{C57}}{bs_{CAST}}$$

where bf is burst frequency and bs is burst size.

### Calculating allelic bias based on simulated observations

For Fig 5, we used the burst kinetics parameters inferred from the C57 allele and simulated observations for each gene twice for a varying number of observations (*n* = 10, 20, 50, 100, 1000 and 10000 observations). We then calculated

$$\max\left(\sum_{k}^{n}B(a_{1_{k}},a_{2_{k}}),\sum_{k}^{n}B(a_{2_{k}},a_{1_{k}})\right)/n$$

for each gene where a_1k_ and a_2k_ are the observed values for the *kth* simulated pair of observations and

$$B(a_{1},a_{2})=\left\{ \begin{array}{c} 1,\;a_{1}>a_{2}\\ 0,\;a_{1}\leq a_{2} \end{array}\right.$$

## Supporting information

S1 Fig Observed fraction of cells with allelic expression patterns compared against those predicted. Predicted fraction of cells with (A) biallelic, (B) no expression, monoallelic expression from the (C) C57 and (D) CAST allele based on either the Poisson model (left) or the two-state model of transcription (right).
(PDF)

S2 Fig Theoretical dependence of bursting parameters on allelic expression patterns. The probabilities of having biallelic, monoallelic (from CAST or C57) or no detectable expression directly predicted from the two-state model of transcription. Inferred kinetics were used for each gene. Plotting the allelic expression patterns as a function of burst frequency (A) and burst size (B). Note, the results are almost identical to the dependencies observed in single-cell RNA-seq data (shown in Fig 2B and 2C).
(PDF)

S3 Fig Investigating the bursting parameter dependence on allelic expression patterns observed on cells *in vivo*. Analyses on the three largest cell type clusters observed in the single-cell RNA-seq analysis of the mouse skin. (top) T-cells (n = 4,299 genes and 83 cells), (middle) Lower Hair Follicle cells (n = 5,807 genes and 75 cells), (bottom) Interfollicular Epidermal cells (n = 5,145 genes and 57 cells). Left panels show the relationship between inferred burst kinetics and allelic expression patterns. Right panels show the correlations between predicted and actual allelic expression patters, with spearman correlation coefficient in the bottom right corners. Note these patterns observed in cells *in vivo* are highly consistent with the analyses performed on cells in primary cultures.
(PDF)

S4 Fig Comparison of ubiquitously expressed and expression matched genes. Histogram and densities showing the ratio (Observed/Expected fraction biallelic expression) for ubiquitously expressed genes and random genes with matched total expression across cells.
(PDF)

S5 Fig Comparison of allelic biased expression to bursting parameters. (A) Histogram showing the distribution of *P*(C57 > CAST | C57 ≠ CAST, n = 7,606 genes). (B) Relationship between burst frequency and equal expression (which is dominated by no expression on either allele).
(PDF)

S1 Table Inferred transcriptional burst parameters for the C57 and CAST allele. k_on, k_off and k_syn are maximum likelihood estimates. bf_lower, bf_upper,bs_lower and bs_upper are confidence intervals based on bootstrap estimates. bf_n and bs_n are the successful number of bootstrap attempts (out of 100).
(XLSX)

S2 Table Predicted probabilities, observed fractions and mean expression for all genes used in the analysis. Analysis code is also available at https://github.com/sandberg-lab/aRME_and_bursting.
(XLSX)
