# Supplementary material for: Transcriptional bursts explain autosomal random monoallelic expression and affect allelic imbalance
Source: PLoS Comput Biol. 2021 Mar 9;17(3):e1008772. doi: 10.1371/journal.pcbi.1008772 (PMC7978379; doi:10.1371/journal.pcbi.1008772)
Supplement: S1 Fig — Predicted fraction of cells with (A) biallelic, (B) no expression, monoallelic expression from the (C) C57 and (D) CAST allele based on either the Poisson model (left) or the two-state model of transcription (right). (PDF) [file pcbi.1008772.s001.pdf]

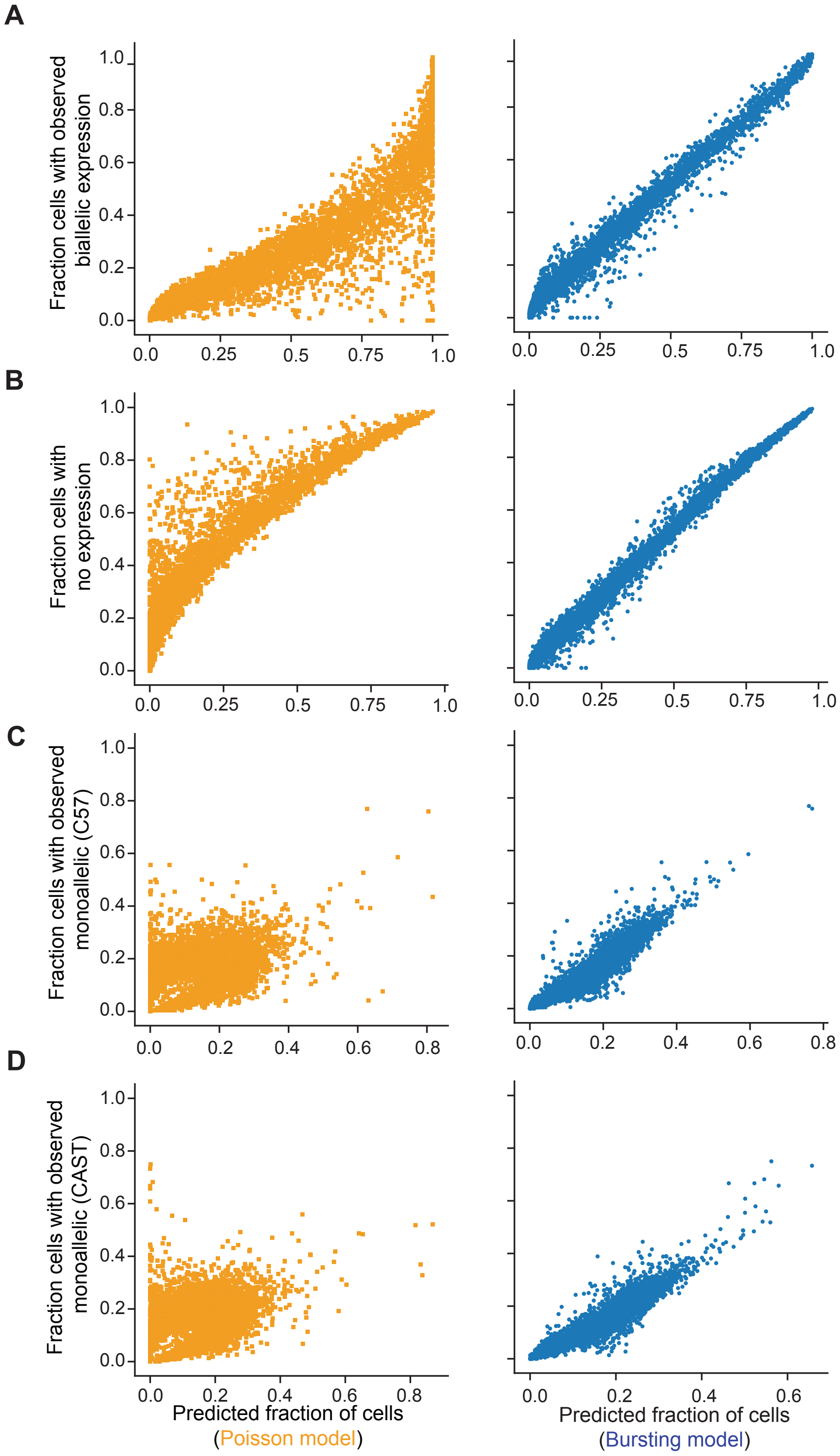

S1 Fig. Observed fraction of cells with allelic expression patterns compared against those predicted.
Predicted fraction of cells with (A) biallelic, (B) no expression, monoallelic expression from the (C) C57 and (D) CAST allele based on either the Poisson model (left) or the two-state model of transcription (right).
