# Supplementary material for: Transcriptional bursts explain autosomal random monoallelic expression and affect allelic imbalance
Source: PLoS Comput Biol. 2021 Mar 9;17(3):e1008772. doi: 10.1371/journal.pcbi.1008772 (PMC7978379; doi:10.1371/journal.pcbi.1008772)
Supplement: S2 Fig — The probabilities of having biallelic, monoallelic (from CAST or C57) or no detectable expression directly predicted from the two-state model of transcription. Inferred kinetics were used for each gene. Plotting the allelic expression patterns as a function of burst frequency (A) and burst size (B). Note, the results are almost identical to the dependencies observed in single-cell RNA-seq data (shown in Fig 2B and 2C). (PDF) [file pcbi.1008772.s002.pdf]

Supplementary Figure 2

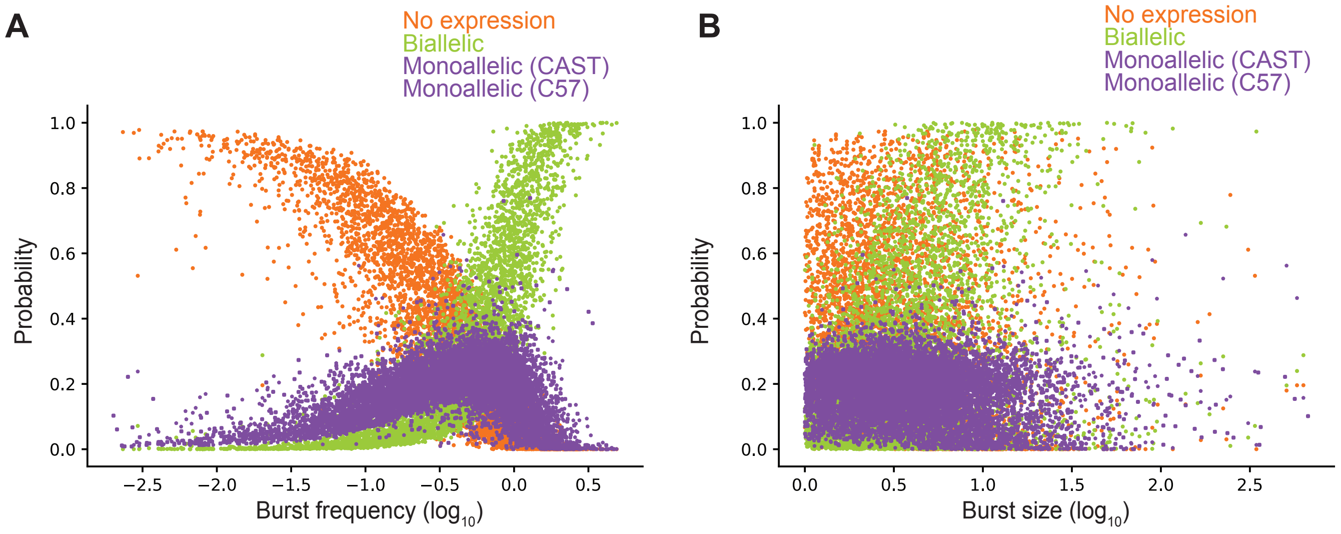

S2 Fig. Theoretical dependence of bursting parameters on allelic expression patterns. The probabilities of having biallelic, monoallelic (from CAST or C57) or no detectable expression directly predicted from the two-state model of transcription. Inferred kinetics were used for each gene. Plotting the allelic expression patterns as a function of burst frequency (A) and burst size (B). Note, the results are almost identical to the dependencies observed in single-cell RNA-seq data (shown in Fig 2B and 2C).
