# Supplementary material for: Transcriptional bursts explain autosomal random monoallelic expression and affect allelic imbalance
Source: PLoS Comput Biol. 2021 Mar 9;17(3):e1008772. doi: 10.1371/journal.pcbi.1008772 (PMC7978379; doi:10.1371/journal.pcbi.1008772)
Supplement: S3 Fig — Analyses on the three largest cell type clusters observed in the single-cell RNA-seq analysis of the mouse skin. (top) T-cells (n = 4,299 genes and 83 cells), (middle) Lower Hair Follicle cells (n = 5,807 genes and 75 cells), (bottom) Interfollicular Epidermal cells (n = 5,145 genes and 57 cells). Left panels show the relationship between inferred burst kinetics and allelic expression patterns. Right panels show the correlations between predicted and actual allelic expression patters, with spearman correlation coefficient in the bottom right corners. Note these patterns observed in cells in vivo are highly consistent with the analyses performed on cells in primary cultures. (PDF) [file pcbi.1008772.s003.pdf]

Supplementary Figure 3

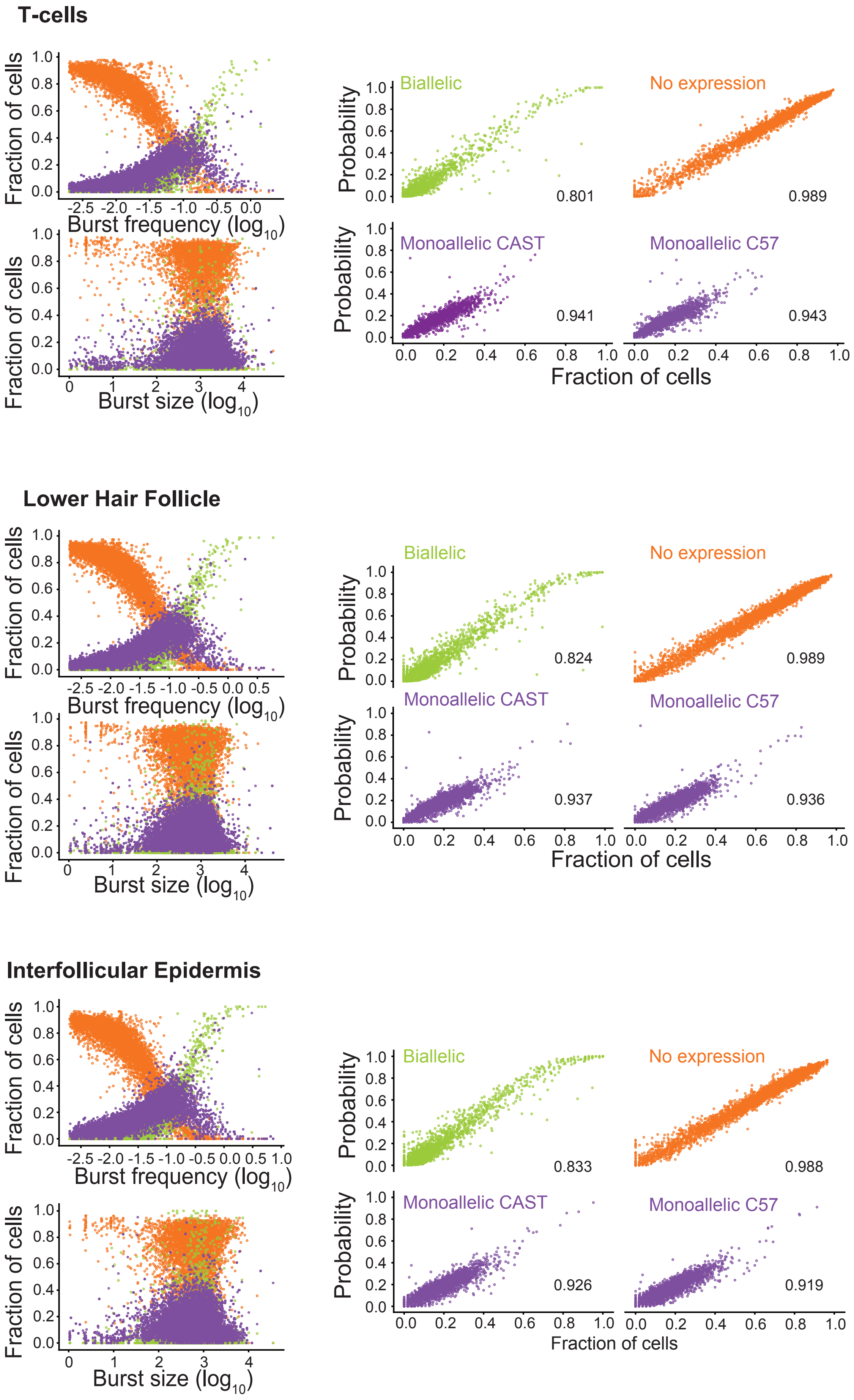

S3 Fig. Investigating the bursting parameter dependence on allelic expression patterns observed on cells in vivo. Analyses on the three largest cell type clusters observed in the single-cell RNA-seq analysis of the mouse skin. (top) T-cells (n = 4,299 genes and 83 cells), (middle) Lower Hair Follicle cells (n = 5,807 genes and 75 cells), (bottom) Interfollicular Epidermal cells (n = 5,145 genes and 57 cells). Left panels show the relationship between inferred burst kinetics and allelic expression patterns. Right panels show the correlations between predicted and actual allelic expression patters, with spearman correlation coefficient in the bottom right corners. Note these patterns observed in cells in vivo are highly consistent with the analyses performed on cells in primary cultures.
