# Supplementary material for: Transcriptional bursts explain autosomal random monoallelic expression and affect allelic imbalance
Source: PLoS Comput Biol. 2021 Mar 9;17(3):e1008772. doi: 10.1371/journal.pcbi.1008772 (PMC7978379; doi:10.1371/journal.pcbi.1008772)
Supplement: S4 Fig — Histogram and densities showing the ratio (Observed/Expected fraction biallelic expression) for ubiquitously expressed genes and random genes with matched total expression across cells. (PDF) [file pcbi.1008772.s004.pdf]

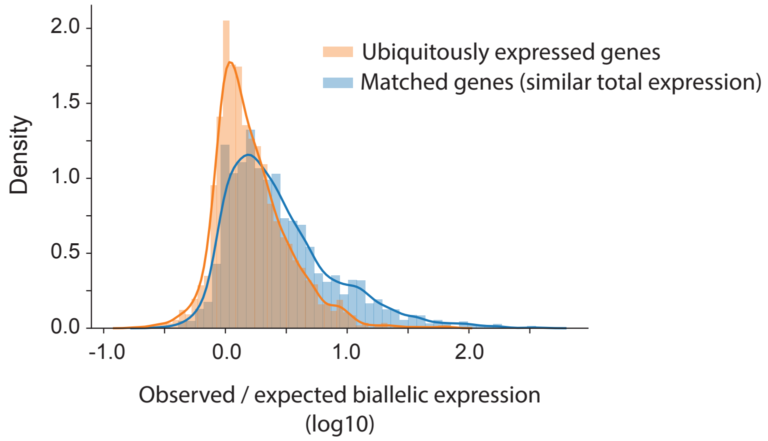

S4 Fig. Comparison of ubiquitously expressed and expression matched genes. Histogram and densities showing the ratio (Observed/Expected fraction biallelic expression) for ubiquitously expressed genes and random genes with matched total expression across cells.
