# Supplementary material for: Transcriptional bursts explain autosomal random monoallelic expression and affect allelic imbalance
Source: PLoS Comput Biol. 2021 Mar 9;17(3):e1008772. doi: 10.1371/journal.pcbi.1008772 (PMC7978379; doi:10.1371/journal.pcbi.1008772)
Supplement: S5 Fig — (A) Histogram showing the distribution of P(C57 > CAST | C57 ≠ CAST, n = 7,606 genes). (B) Relationship between burst frequency and equal expression (which is dominated by no expression on either allele). (PDF) [file pcbi.1008772.s005.pdf]

Supplementary Figure 5

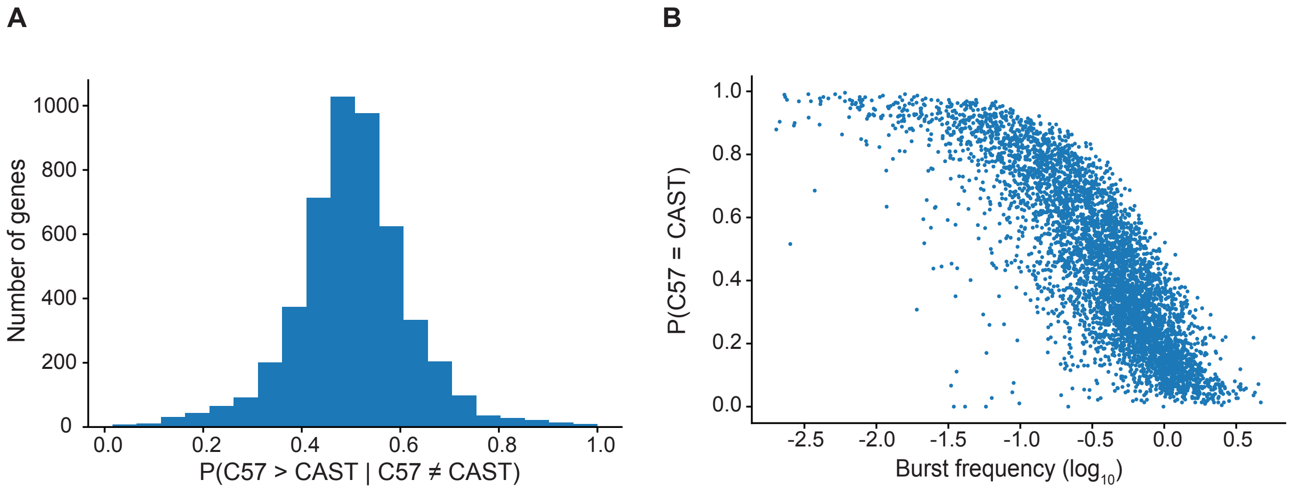

S5 Fig. Comparison of allelic biased expression to bursting parameters.
(A) Histogram showing the distribution of P(C57 > CAST | C57 ≠ CAST, n = 7,606 genes). (B) Relationship between burst frequency and equal expression (which is dominated by no expression on either allele).
